# Emotional meanings attributed to sexual experiences reported by Brazilian Men with Head and Neck Cancer seen at a Public University Oncologic Outpatient Service: A Qualitative Study

**DOI:** 10.1192/j.eurpsy.2024.1341

**Published:** 2024-08-27

**Authors:** R. S. E. Sant’Ana, C. S. P. Lima, E. R. Turato

**Affiliations:** Lab of Clinical-Qualitative Research, University of Campinas, Campinas, Brazil

## Abstract

**Introduction:**

“What can and what cannot I do in a sexual relationship?” |In this way, a middle-aged man with HNC - Head and Neck Cancer, under chemotherapy or radiotherapy, asks. This doubt is raised to the oncologist, radiotherapist, nurse or psychotherapist. Apparently, his concern is objective, considering he has a severe diagnosis and important treatments. However, underlying the manifest doubt, there are symbolic constructions in his mind that generate anguish. The health professionals’ response must go beyond information. They also must understand the symbolic emotional meanings associated with the patient’s speech. This attitude will bring a psychotherapeutic effect to the ill man.

**Objectives:**

To interpret symbolically sexual and emotional experiences reported by male patients diagnosed with HNC under outpatient treatment in a public specialized clinical unit.

**Methods:**

We used the CQM - Clinical-Qualitative Method (Turato. Portuguese Psychos. J, 2000 2(1): 93-108). For data collection, the main researcher used the Semi-Directed Interview with Open-ended Questions In-Depth and Field Notes. The employ of the Seven Steps of the CQCA - Clinical-Qualitative Content Analysis (Faria-Schützer et al. Cien Saude Colet. 2021; 26(1): 265-274) brings us to discussion categories. The sample was closed with 12 patients according to the information saturation strategy (Fontanella et al. Cad Saude Publica. 2008; 24(1): 17-27). The interviews were conducted by the first author of this abstract, a male nurse, as part of his master’s research at a postgraduate course in Oncology. The findings were validated by peer reviewers from the Lab of Clinical-Qualitative Research at the State University of Campinas.

**Results:**

Two categories were chosen for this presentation: ‘The dyad perceived in the felt body and the experienced body’, and ‘The body re-signified between the sexual and affective dimensions’. The body symbolized before and after the illness experiences a movement in phenomenological consciousness that leads to external changes in its attitudes. The patient needs now to ask himself and others what this body can - or cannot - do. The severely ill body imposes new meanings for life and sexuality. It does not cancel the wish but asks for a new channelling of your psychic/sexual energies.

**Conclusions:**

These findings indicate that patients with HNC want to talk about sexuality and ask about the risks of sexual activity, contrary to what the common view supposes. Traditional Balint groups met with the multidisciplinary team can be beneficial for doctors and nurses to deal with their own emotional limitations. Furthermore, the Consultation-Liaison Psychiatry, under the approach of psychosomatic medicine, focuses on the care of patients with behavioural and emotional manifestations, together with the work of the oncologists.

**Disclosure of Interest:**

None Declared

